# Rationale and design of the oral HEMe iron polypeptide Against Treatment with Oral Controlled Release Iron Tablets trial for the correction of anaemia in peritoneal dialysis patients (HEMATOCRIT trial)

**DOI:** 10.1186/1471-2369-10-20

**Published:** 2009-07-28

**Authors:** Katherine A Barraclough, Euan Noble, Diana Leary, Fiona Brown, Carmel M Hawley, Scott B Campbell, Nicole M Isbel, David W Mudge, Carolyn L van Eps, Joanna M Sturtevant, David W Johnson

**Affiliations:** 1 Department of Nephrology, University of Queensland at Princess Alexandra Hospital, Brisbane, Australia; 2 Department of Nephrology, Monash Medical Centre, Melbourne, Australia; 3 Australasian Kidney Trials Network, School of Population Health, University of Queensland, Brisbane Australia

## Abstract

**Background:**

The main hypothesis of this study is that oral heme iron polypeptide (HIP; Proferrin^® ^ES) administration will more effectively augment iron stores in erythropoietic stimulatory agent (ESA)-treated peritoneal dialysis (PD) patients than conventional oral iron supplementation (Ferrogradumet^®^).

**Methods:**

Inclusion criteria are peritoneal dialysis patients treated with darbepoietin alpha (DPO; Aranesp^®^, Amgen) for ≥ 1 month. Patients will be randomized 1:1 to receive either slow-release ferrous sulphate (1 tablet twice daily; control) or HIP (1 tablet twice daily) for a period of 6 months. The study will follow an open-label design but outcome assessors will be blinded to study treatment. During the 6-month study period, haemoglobin levels will be measured monthly and iron studies (including transferring saturation [TSAT] measurements) will be performed bi-monthly. The primary outcome measure will be the difference in TSAT levels between the 2 groups at the end of the 6 month study period, adjusted for baseline values using analysis of covariance (ANCOVA). Secondary outcome measures will include serum ferritin concentration, haemoglobin level, DPO dosage, Key's index (DPO dosage divided by haemoglobin concentration), and occurrence of adverse events (especially gastrointestinal adverse events).

**Discussion:**

This investigator-initiated multicentre study has been designed to provide evidence to help nephrologists and their peritoneal dialysis patients determine whether HIP administration more effectively augments iron stores in ESP-treated PD patients than conventional oral iron supplementation.

**Trial Registration:**

Australia New Zealand Clinical Trials Registry number ACTRN12609000432213.

## Background

Anemia develops early in the course of chronic kidney disease (CKD) and is nearly universal in patients with CKD stage 5 [1]. The severity of anemia is related to both the degree of loss of glomerular filtration rate (GFR) and the cause of kidney disease [2]. The development of erythropoietic stimulatory agents (ESA), such as recombinant human erythropoietin (EPO) and darbepoietin alpha (DPO), has resulted in substantial health benefits for patients with end-stage renal failure, including improved quality of life, reduced blood transfusion requirements, decreased left ventricular mass, diminished sleep disturbance and enhanced exercise capacity [3,4]. Unfortunately, a considerable proportion of such patients exhibit a suboptimal haematologic response to ESA, which in most cases is due to inadequate iron supply to the erythron [1]. Concomitant iron supplementation is therefore required in as many as 90% of EPO-treated individuals [5].

In haemodialysis populations, several investigations, including a randomised controlled trial, have consistently demonstrated that intravenous iron supplementation is superior to oral iron replacement with respect to enhancing body iron stores, augmenting haemoglobin levels and reducing EPO requirements [5-7]. However, there is ongoing controversy as to whether iron supplementation is best administered orally or intravenously in peritoneal dialysis (PD) patients (and pre-dialysis patients), in whom repeated intravenous cannulation is logistically more difficult. Our group recently performed a cross-over trial of oral iron versus 2-monthly intravenous iron infusions in 28 PD patients and demonstrated that IV iron supplementation was associated with a much lower incidence of gastrointestinal disturbances (11% versus 46%, p < 0.05) and superior haemoglobin levels and body iron stores, but exceeded the cost of oral iron treatment by 6.5-fold [8]. Other investigators have similarly demonstrated that oral iron supplements are unable to maintain adequate iron stores in PD patients over medium-to-long term periods [5], principally because of poor compliance, gastrointestinal side effects, suboptimal gastrointestinal absorption of iron (particularly in the presence of an acute phase response and high ferritin levels), and medication costs [9]. In spite of this evidence, many authors [10-12] and clinical practice guidelines [13-15] have recommended oral iron supplementation for PD patients in the first instance, because of its greater simplicity, lower cost and avoidance of the need for repeated intravenous cannulation.

The development of an oral iron supplement which safely, cheaply and more effectively maintains iron stores and haemoglobin levels in ESP-treated PD patients would therefore be of considerable clinical utility. Preliminary evidence suggests that heme iron polypeptide (HIP) may represent such a promising, novel, therapeutic strategy. HIP is produced by hydrolysis of bovine haemoglobin resulting in a highly soluble heme moiety that contains more than 1% iron. Since heme is absorbed via a different receptor to that utilised by nonheme (ionic) iron [16,17], the absorption kinetics and gastrointestinal side effect profiles of HIP and ionic iron are dissimilar. Administration of HIP to 14 healthy subjects was associated with fewer side effects and significantly higher bioavailability compared with nonheme iron [18]. Specifically, the study demonstrated that HIP increased serum iron levels 23 times greater than ferrous fumarate on a milligram-per-milligram basis. One of the possible reasons for why heme iron is more bioavailable than ionic iron is thought to be due the fact that the gastrointestinal absorption of heme iron is far less affected by dietary constituents, such as polyphenolic tannins, phytates, soy and dairy products [18]. Hallberg et al [19] also demonstrated that the absorption of heme iron may be more than 10 times greater than that of iron salts in subjects with serum ferritin levels greater than 400 ng/mL (898 pmol/L). Moreover, the gastrointestinal absorption of both heme and non-heme iron is significantly increased by stimulation with ESA [20]. Recently, Nissenson et al [21] performed an open-label, pre-test/post-test trial of HIP (1 tablet tds) administered in lieu of intravenous iron supplementation to 37 ESA-treated haemodialysis patients over a 6 month period. Although 4 (11%) of 37 patients dropped out due to gastrointestinal intolerance (n = 3) or insufficient iron supplementation (n = 1) and 5 patients (14%) were excluded due to unrelated complications or protocol violation, HIP successfully replaced IV therapy in the majority of patients, resulting in maintenance of haematocrit targets and iron stores and significant improvement in EPO efficiency (mean EPO dose/haemoglobin levels fell from 1270 to 1023 U.g/mo/dL, p = 0.04). However, the results of this study were significantly limited by its open label non-randomised design, potential for co-intervention and observer biases, high drop-out rate (25% over 6 months) and failure to analyse on an intention to treat basis. There have been no other trials of HIP in chronic kidney disease patients (including PD patients).

## Methods/Design

### Objectives

The primary objective is to determine whether oral HIP administration (two tablets nocte per os, equivalent to 24 mg elemental iron per day) results in significantly higher transferrin saturation (TSAT) values in PD patients treated with DPO at 6 months compared with conventional oral slow-release ferrous sulphate supplementation (two tablets nocte per os, equivalent to 210 mg elemental iron per day).

The secondary objectives will be to determine whether oral HIP administration (two tablets nocte per os, equivalent to 24 mg elemental iron per day) to DPO-treated PD patients over 6 months results in

a) an increase in serum ferritin concentrations;

b) an increase in haemoglobin levels;

c) a reduction in the prescribed dosages of DPO (Aranesp^®^, Amgen);

d) a reduction in Key's index (DPO dosage divided by haemoglobin concentration); and,

e) a reduction in significant side effects (especially gastrointestinal side effects).

Ethics approval for the **HEM**e iron polypeptide **A**gainst **T**reatment with **O**ral **C**ontrolled **R**elease **I**ron **T**ablets trial (HEMATOCRIT trial) has been obtained from the local Institutional Ethics Committee in all participating centres prior to study initiation and patient enrolment. The study will be performed in accordance with the 2000 Edinburgh, Scotland Revision of the Declaration of Helsinki, the National Health and Medical Research Committee (NHMRC) Statement on Human Experimentation, Joint NHMRC/AVCC Statement and Guidelines on Research Practice, applicable ICH guidelines and the Therapeutic Goods Administration (TGA) – Note for guidance on good clinical practice (CPMP/ICH/135/95) annotated with TGA.

### Participants

The study population includes adult (18 years or over) patients willing to give informed consent who have been on PD for > 1 month and receiving DPO for > 1 month. Patients will be recruited by local investigators from PD units at three Australian centres. The clinical and demographic characteristics of this population are comparable to those reported for the Australian and New Zealand PD population by the ANZDATA Registry. This, along with the multicentre nature of the trial will enhance its generalisability.

Exclusion criteria include:

1. Patients with a history of psychological illness or condition which interferes with their ability to understand or comply with the requirements of the study.

2. Pregnancy or breast-feeding.

3. Known hypersensitivity to, or intolerance of, oral iron, HIP or DPO.

4. Active peptic ulcer disease.

5. Vitamin B12 or folate deficiency.

6. Recent (within 1 month) acute infection.

7. Parathyroid hormone level > 100 pmol/L.

8. Serum aluminium > 2 μmol/L.

9. Presence of systemic haematological disease (including antibody-mediated pure red cell aplasia) or known haemoglobinopathy

10. Major surgery, infection, acute myocardial infarction or malignancy within the last 3 months.

11. Intravenous iron therapy, vitamin C therapy, melatonin treatment, androgen therapy or blood transfusion within the previous month.

12. Serum ferritin ≥ 500 μg/mL or transferrin saturation (TSAT) ≥ 50%.

13. Religious or other objection to consuming product prepared from bovine blood.

### Study design

**Figure 1:**
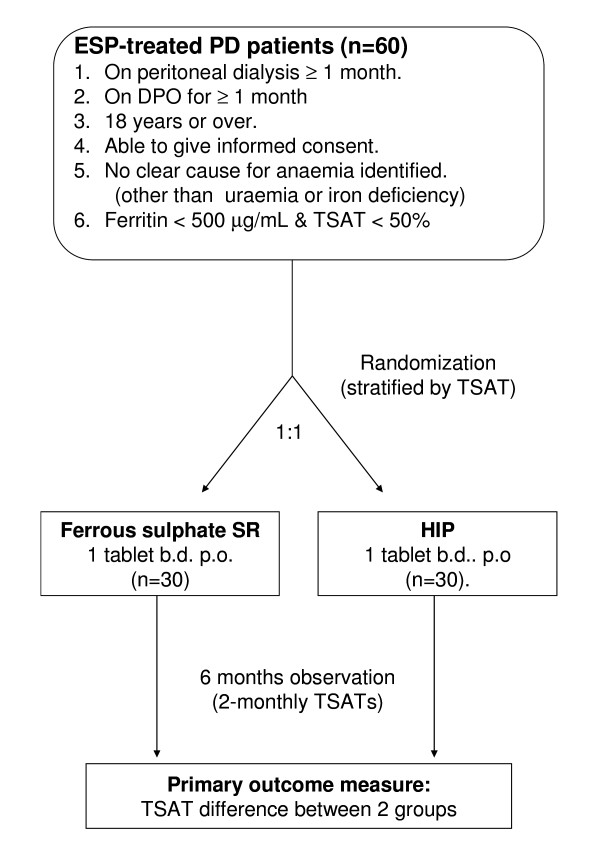
**Trial schema**.

The study is a prospective, open-label, randomised controlled trial. Patients will be randomised to one of two treatment groups in equal proportion (Figure 1). To ensure adequate concealment of allocation, the randomisation will be performed using sequentially numbered, opaque, sealed envelopes, stratified for the presence or absence of a TSAT ≤ 20%. The sequence of interventions was obtained from a computer-generated random number list in permuted blocks provided through the Australasian Kidney Trials (AKTN) network.

### Experimental Intervention

HIP (Proferrin^®^ES, Colorado Biolabs, USA) is approved for use as a dietary/nutritional supplement in Canada and the United States of America. The usual dose is 1 tablet (equivalent to 12 mg elemental iron) taken twice or thrice daily, either with or without food. The side effects reported with HIP are similar to those associated with conventional oral iron preparations and include nausea, vomiting and constipation. However, trials in healthy individuals [18] and in haemodialysis patients [21] suggest that the incidence of such adverse drug reactions is relatively low, possibly due to the different absorption characteristics of heme versus non-heme iron. For example, in a study of 37 haemodialysis patients receiving HIP for 6 months, constipation occurred in 3 (8%) patients and was the only observed adverse effect of the agent. The dose of HIP used in that investigation (3 tablets per day) was 50% higher than that proposed in the present study (2 tablets per day). There are no other published data concerning the side effect profile of HIP in end-stage renal failure patients. Concern has previously been expressed about the possibility of transmission of bovine spongiform encephalopathy (BSE) through the consumption of bovine tissue [21]. Currently, HIP is manufactured from red blood cells of cows of American origin, and both the US Department of Agriculture and Food and Drug Administration (FDA) currently maintain that the United States is free of BSE. In addition, the putative infectious agents for BSE, conformationally shifted neuronal membrane copper-binding proteins called prions, usually are not found in blood [21]. Thus, the risk of BSE from taking HIP is negligible.

### Control Intervention

Slow-release ferrous sulphate (Ferrogradumet^®^, Abbott, Sydney, Australia) is registered in Australia for use as an oral iron supplement and is one of the most common supplements prescribed in dialysis populations. The usual recommended dose is 2 to 3 tablets a day (equivalent to 210 to 315 mg of elemental iron per day). However, gastrointestinal side effects (especially constipation) usually limit the maximum dosage in end-stage renal failure patients to 2 tablets per day (the dose proposed in the current study). The side effects reported with Ferro-Gradumet are similar to those associated with other conventional oral iron preparations, although the incidence may be lower due to the controlled release nature of the formulation. They are as follows: nausea, vomiting, abdominal pain or discomfort, blackening of stools, diarrhoea and constipation. In a study of 28 PD patients at Princess Alexandra Hospital, oral Ferrogradumet^® ^administration in a dose of 2 tablets per day for 4 months was associated with significant gastrointestinal side effects in 46% of patients (constipation 38%, nausea 19%, abdominal pain 4%). A randomised, placebo-controlled trial of oral ferrous sulphate in 32 consecutive iron-replete dialysis patients similarly revealed significant gastrointestinal side effects in 50% of subjects [22]. Ferrous sulphate absorption is significantly inhibited by concomitant food and phosphate binder medications. Consequently, subjects participating in the present trial will be instructed to take their study medications on an empty stomach and at least 2 hours apart from phosphate binder ingestion.

### Concurrent Treatments

Vitamin B and folic acid supplementation are permitted. Vitamin C supplementation, melatonin and androgen therapy are prohibited during the study period.

### Blinding

Blinding is not able to be performed as it is not possible to formulate identical-appearing preparations of HIP and slow-release iron. The controlled release iron tablets are too large to enclose in capsules and cannot be cut or ground. Consequently, the study will follow an open label design. An individual patient's participation in the study will cease at the end of the 6 month study period. If, before this time, the patient experiences severe anaemia (<65 g/L), symptomatic anaemia or the patient's attending physician believes that additional therapy is required (eg blood transfusion), they will be considered to have reached an end-point and will be withdrawn from study medication, but will still be followed up, with outcomes measured.

### Outcome measures

The primary outcome measure will be the difference in TSAT values between the HIP and ferrous sulphate groups at the end of the 6 month study period.

The secondary outcome measures will be the differences between the 2 groups at the end of the 6 month study period with respect to serum ferritin concentration, haemoglobin level, DPO dosage, Key's index (DPO dosage divided by haemoglobin) and incidence of adverse events.

### Procedures

**Figure 2:**
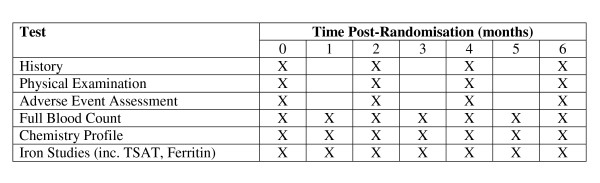
**Trial schedule (all tests are ordered as per routine clinical practice)**.

Patients will take 2 tablets (HIP or ferrous sulphate) nocte for a period of 6 months. They will be instructed to take their study medications on an empty stomach and at least 2 hours apart from phosphate binder ingestion. At the time of inclusion in the study, demographic and clinical data will be recorded. All patients will be allowed their usual diet during the study period. DPO dosages will modified according to unit protocols, based on haemoglobin level. Full blood counts will be measured monthly, as per usual clinical practice. Iron studies will be measured every 2 months, as usual clinical practice. Patients will receive a medical review by their nephrologists every 2 months, as per usual clinical practice. No other procedure will be undertaken and all other care (including blood tests) will be provided according to standard unit protocols. Figure 2 outlines the trial schedule.

An individual patient's participation in the study will cease at the end of the 6 month study period. If, before this time, the patient experiences severe anaemia (<65 g/L), symptomatic anaemia (e.g. new onset or worsening shortness of breath or ischaemic chest pain attributable to the low haemoglobin) or the patient's attending physician believes that additional therapy is required (eg blood transfusion), they will be considered to have reached an end-point and will be withdrawn from study medication, but will still have blood counts measured monthly and followed for study outcomes. A patient's participation in the study will also cease if, during the course of the study, they complete PD therapy (eg renal transplantation or conversion to haemodialysis).

### Adverse Events

The number and proportion of subjects who report treatment-emergent adverse events will be summarized for each treatment group. Treatment emergent events include events that start on or after Day 0 of the study [that is the first day of Study Drug administration], and were not present at baseline, or were present at baseline, but increased in severity after the start of the study. The Medical Dictionary for Regulatory Activities [MedDRA] Terminology will be used to classify all adverse events with rESAect to System Organ Class [SOC], high level group term (HLGT), and preferred term.

For the purposes of this study, anaemia (serum Hgb or Hct below the normal range or worsened from baseline) will not be considered an adverse event unless it reaches a severe level (<65 g/L) or is associated with significant symptoms. Similarly, iron deficiency (iron indices below the normal range or worsened from baseline) will not be considered an adverse event.

### Sample size calculations

Prospective power calculations indicate that the study will have adequate statistical power (80% probability) to detect a clinically significant increase in TSAT of 7%, assuming α = 0.05 and a population standard deviation of 8%, if 44 patients were recruited in the study (22 in each group). Allowing for a 15% drop-out rate over 6 months using the adjustment formula of 1/[1 - drop-out rate] ^2^, we aim to recruit a total of 60 patients (30 in each group). We anticipate that a recruitment period of 18 months will be required. The assumptions for these power calculations were based on a previous randomised controlled trial of oral versus intravenous iron supplementation in PD patients [8], in which the baseline values were 107 ± 3 g/L for haemoglobin, 24.2 ± 1.7% for transferrin saturation and 323 ± 46 μg/L for serum ferritin concentration. Based on these assumptions, we estimate the power of the study for secondary outcome measures to be 77% for serum ferritin (δ 150 μg/mL, σ 180 μg/mL), 62% for haemoglobin (δ 7 g/L, σ 10 g/L), 22% for DPO divided by haemoglobin (δ 0.00075 μg.L/kg.g/week, σ 0.002 μg.L/kg.g/week) and 93% for the occurrence of gastrointestinal events (HIP 8% vs ferrous sulphate 46%).

The assumptions used for the power calculations are conservative and based on our previous study in 28 PD patients [22] and that of Nissenson et al in 37 haemodialysis patients ^1^. Because of power considerations and the relatively short duration of the study, no interim analysis is planned.

### Statistical analysis

#### Primary Outcome Analyses

Differences between the intervention and control groups with respect to the primary outcome measure (TSAT level at 6 months) will be measured by comparison of the mean TSAT in each group, adjusted for baseline values (analysis of covariance). Data will be analysed on an intention to treat basis. For patients withdrawing before the end of the 6 month study, their TSAT at the time of withdrawal will be taken as their final haemoglobin.

#### Secondary Outcome Analyses

Secondary analysis will be performed by repeated measures analysis of covariance with and without adjustment for baseline characteristics. The analyses used for secondary outcome measures will include unpaired t-test (serum ferritin, Key's index), Mann-Whitney U-test (DPO dosage) and chi square test (adverse drug reactions).

Categorical baseline characteristics (e.g. sex, race, comorbid illnesses, etc.) will be summarized with the number and percent of subjects in each treatment group with the characteristic. Quantitative characteristics (e.g., age and weight) will be summarized by mean and standard deviation or median [interquartile range], depending on data distribution. The number and percent of subjects who are randomised, treated with randomized Study Drug, require intervention, prematurely discontinue, and complete the study will be summarized. Fisher's exact test or the Chi-square test will be used to assess treatment group differences in the proportions of subjects who require intervention and who complete the study. The number and percent of subjects will be summarised for each reason for premature discontinuation.

## Discussion

This investigator-initiated multicentre Australian study has been designed to provide evidence to help nephrologists and their CKD patients better determine whether HIP (Proferrin^®^ES) will more effectively augment iron stores in ESP-treated PD patients than conventional oral iron supplementation. Concomitant iron supplementation is required in as many as 90% of ESP-treated individuals [5]. However, the optimal means of administering iron to PD patients remains unclear: administration of intravenous iron is logistically difficult and costly, while conventional oral iron supplementation is also associated with poor compliance, gastrointestinal side effects and suboptimal GI absorption of iron [9]. Thus, the development of an oral iron supplement which is able to safely, cheaply and more effectively maintain iron stores and haemoglobin levels in ESP-treated PD patients would fulfil a currently unmet clinical need.

The multicentre nature of this trial will greatly enhance its generalisability. Moreover, the trial sample size has been carefully and prospectively calculated using a minimum clinically important difference in TSAT of 7% and realistic estimates of trial drop-out and non-compliance rates to minimise the risk of a type 2 statistical error.

It is hoped that results will be available in 2010. Demonstration of a significant improvement in iron stores with oral HIP therapy will provide clinicians with an important new therapy for effectively combating inadequate iron stores in PD patients.

## List of abbreviations

ANCOVA: Analysis of covariance; AKTN: Australasian Kidney Trials Network; BSE: Bovine spongiform encephalopathy; CKD: Chronic kidney disease; CTN: Clinical trials notification; DPO: Darbepoetin alpha; EPO: Erythropoietin; ESA: Erythropoiesis stimulating protein; ESKD: End-stage kidney disease; Hct: Haematocrit; HEMATOCRIT: **HEM**e iron polypeptide **A**gainst **T**reatment with **O**ral **C**ontrolled **R**elease **I**ron **T**ablets; Hgb: Haemoglobin; HIP: Heme iron polypeptide; HLGT: High level group term; IVRS: Interactive voice response system; MedDRA: Medical dictionary for regulatory activities; PD: Peritoneal dialysis; SOC: System organ class; TGA: Therapeutic Goods Administration; TSAT: Transferrin saturation.

## Competing interests

### Financial Competing Interests

The study is funded by a grant from Amgen Australia.

DWJ has received consultancy fees and speaker's honoraria from Sanofi-Aventis. He has also received consultancy fees, speaker's honoraria, research grants and conference travel sponsorships from Amgen, Janssen-Cilag and Roche (all manufacturers of erythropoiesis stimulating agents). KB has received conference travel sponsorship from Amgen. FB has received speakers honorarium from Janssen-Cilag and conference travel sponsorship from Amgen and Roche. CMH has received conference travel sponsorships from Amgen and Janssen-Cilag. JS has received speaker's honoraria and conference travel sponsorships from Amgen. The remaining authors declare that they have no financial competing interests.

### Non-Financial Competing interests

The authors declare that they have no non-financial competing interests.

## Authors' contributions

KB participated in trial design and co-ordination, helped draft the manuscript and read and approved the final manuscript. FB participated in trial design and co-ordination and read and approved the final manuscript. EN participated in trial design and co-ordination and read and approved the final manuscript. CH participated in trial design and co-ordination and read and approved the final manuscript. SC participated in trial design and co-ordination and read and approved the final manuscript. NI participated in trial design and co-ordination and read and approved the final manuscript. DM participated in trial design and co-ordination and read and approved the final manuscript. CvE participated in trial design and co-ordination and read and approved the final manuscript. JS participated in trial design and co-ordination and read and approved the final manuscript. DJ, principal Investigator, conceived the study, participated in trial design and co-ordination, helped to draft the manuscript and read and approved the final manuscript. All authors read and approved the final version of the manuscript.

## Pre-publication history

The pre-publication history for this paper can be accessed here:

http://www.biomedcentral.com/1471-2369/10/20/prepub
